# Biomarker value of plasma endothelial microvesicle-derived circRNA 0006222 in vascular ageing and carotid atherosclerosis

**DOI:** 10.3389/fnins.2026.1872315

**Published:** 2026-07-15

**Authors:** Huiting Zhang, Ye Ye, Xiaobing Xu, Dinghua Xu, Jiawu Fu, Yan Wang, Yanfang Chen, Xiaotang Ma

**Affiliations:** 1 Guangdong Key Laboratory of Age-Related Cardiac and Cerebral Diseases, Department of Neurology, Institute of Neurology, Affiliated Hospital of Guangdong Medical University, Zhanjiang, China; 2 Guangdong Mental Health Center, Guangdong Provincial People’s Hospital (Guangdong Academy of Medical Sciences), Southern Medical University, Guangzhou, China; 3 Second Affiliated Hospital of Guangdong Medical University, Zhanjiang, China

**Keywords:** biomarker, carotid atherosclerosis, circular RNAs 0006222, endothelial microvesicles, vascular ageing

## Abstract

**Background:**

Endothelial microvesicles (EMVs) carrying circular RNAs (circRNAs) have emerged as promising novel biomarkers of ageing-associated disorders. This study investigated the potential of plasma EMVs-circ_0006222 as vascular ageing (VA) and carotid atherosclerosis (CAS) biomarkers.

**Methods:**

A circRNA microarray was applied to screen for differentially expressed circRNAs in plasma EMVs obtained from CAS participants, VA participants and healthy controls. VA was assessed according to intima thickness, and CAS was evaluated by using computed tomography angiography. The plasma EMVs-circ_0006222 level was determined via qRT-PCR. EMVs-circ_0006222 levels in patients of different ages and with different severities of CAS were compared. The diagnostic potential of EMVs-circ_0006222 was evaluated by using multivariable logistic regression and receiver-operator characteristic (ROC) curve analyses.

**Results:**

A total of 47 healthy controls, 81 VA participants and 216 CAS participants were included in this study. Plasma EMVs-circ_0006222 levels were observed to be negatively associated with age. The plasma level of EMVs-circ_0006222 was markedly reduced in elderly participants, as well as VA and CAS participants; moreover, the decrease was most evident in the CAS group. The plasma EMVs-circ_0006222 level was declined in the severe CAS compared with the mild and moderate CAS subjects. Plasma EMVs-circ_0006222 level was identified by multivariate logistic regression as a significant, independent risk factor for VA and CAS. The areas under the ROC curve of plasma EMVs-circ_0006222 for diagnosing VA and CAS were 0.628 and 0.773, respectively.

**Conclusion:**

Plasma EMVs-circ_0006222 may serve as a promising biomarker for VA and CAS.

## 1 Introduction

Carotid atherosclerosis (CAS) is among the main causes of ischaemic stroke and remains latent and asymptomatic during the initial and aggravation stages of the condition (Abbott, 2021). Approximately 18–25% of all ischaemic strokes are attributable to CAS (školoudík et al., 2022). In the early stage, carotid atherosclerosis (CAS) is marked by augmented carotid intima-media thickness (IMT), whereas advanced CAS is characterized by arterial stiffness, and the development of carotid plaques and luminal stenosis (Zhou et al., 2026). These features are strongly associated with vascular aging (VA), which is considered as a risk factor for CAS (Mitchell et al., 2018; Zhang et al., 2024). The annual progression rate to more severe carotid stenosis among individuals with VA is approximately 9.3% (Muluk et al., 1999) and the 5-year ipsilateral stroke risk for individuals with 70–99% stenosis varies from 14.6 to 18.3% (Howard et al., 2021). Consequently, the investigation of approaches for the early identification and management of VA and CAS holds significant value.

Currently, IMT is primarily assessed by using colour Doppler sonography (CDS) and computed tomography angiography (CTA) (Herraiz-Adillo et al., 2026; Nan et al., 2026). However, both techniques demonstrate limitations. For example, CDS could be compromised by the presence of bones, thereby diminishing image quality and diagnostic accuracy (Arning and Eckert, 2004; Collado et al., 2024). The radioactive contrast agent used in CTA may pose various risks such as nephropathy and anaphylaxis (Morello et al., 2018) and is susceptible to metal-related streak artefacts (Núñez et al., 2004). Moreover, the operator-independent nature of CDS and CTA may result in inconsistent results. The exploration of a blood-based biomarker to evaluate VA and CAS may represent a more personalised strategy. Biochemical indicators exemplified by the neutrophil-lymphocyte index and platelet-lymphocyte index and extracellular matrix metalloproteinase have demonstrated predictive value for atherosclerotic events, with areas under the curve ranging from 0.67 to 0.79 (Kampf et al., 2023; Liao et al., 2024). However, these indicators could be influenced by other inflammatory or haematological conditions. Therefore, we aimed to investigate more objective biomarkers exhibiting greater diagnostic efficacy for VA and CAS.

Endothelial dysfunction is a key mediator in the pathophysiology of atherosclerosis, it can drive age-related arterial stiffness, which impairs arterial function, exacerbates pulsatile hemodynamic stress, and further accelerates atherosclerotic plaque formation in carotid vessels (Zhou et al., 2024; Zhai et al., 2026). Endothelial microvesicles (EMVs) are released by activated or dying endothelial cells. Elevated levels of EMVs are detectable in the circulation before the appearance of clinical symptoms (Khalfaoui et al., 2025), with studies demonstrating significantly increased EMVs concentrations in patients with diabetes and in those with previous occurrences of acute myocardial infarction for several weeks (Heitmar and Blann, 2022; Zhang et al., 2025). Moreover, EMVs carry various types of cargo (such as mRNAs, miRNAs and circRNAs) from parent cells. These cargo molecules are highly specialised, thus precisely mirroring pathogenic alterations in their parent cells (Hernández-López et al., 2025). Additionally, RNA molecules remain remarkably stable within the vesicular structure, as the membrane effectively protects them from degradation by circulating RNases, thereby maintaining their integrity even under varying temperature and pH conditions (Cheng et al., 2014). These findings indicate that EMVs and their RNA cargo can reflect early endothelial activation and damage, thus serving as promising indicators of vascular pathology. Several studies in acute ischaemic stroke patients have demonstrated marked dysregulation of circRNAs in plasma brain-derived exosomes, with five circRNAs being upregulated and correlated with neurological deficits (Jiang et al., 2024). CircBTBD7-420aa, which is encoded by hsa_circ_0000563, can inhibit atherosclerosis progression via exosome-mediated delivery (Gan et al., 2025). The specific role of EMV-derived circRNAs in VA and CAS pathogenesis remains largely unexplored. In this study, our circRNA microarray results revealed decreased plasma EMVs-circ_0006222 levels in VA and CAS participants. Gene prediction indicated that the deSUMOylation enzyme known as sentrin-specific protease 1 (SENP1) serves as a potential target gene for EMVs-circ_0006222. SENP1 has been implicated in the regulation of lipid metabolism, cardiovascular function, oxidative stress, and inflammation (Zheng et al., 2020). Emerging findings has demonstrated that oxidative stress, lipid metabolism, and inflammatory responses are closely related to CAS and VA. Thus, we hypothesized that EMVs-circ_0006222 may be a potential biomarker for CAS and VA.

The present study sought to explore the correlations between plasma EMVs-circ_0006222 levels and both VA and CAS by investigating clinical peripheral blood samples, which may offer novel perspectives on the early detection and understanding of VA and CAS.

## 2 Materials and methods

### 2.1 Study subjects and enrolment criteria

From September 2021 to October 2023, subjects were recruited at the Affiliated Hospital of Guangdong Medical University. The following inclusion criteria were utilized for VA and CAS patients: (1) male or female participants aged more than 18 years and (2) VA and CAS diagnosed via carotid Doppler ultrasound and CTA, respectively. The exclusion criteria were as follows: (1) haemorrhage or other diseases (such as tumour, arteriovenous malformations, stroke, and brain abscesses) detected on CT/MRI at admission; (2) myocardial infarction, congestive heart failure, and atrial fibrillation; and (3) pregnant or lactating women. Subjects were recruited and assigned to the healthy control, VA and CAS groups. The above mentioned groups were further subdivided into young (18–45-years-old), middle-aged (46–65-years-old), and aged (> 65-years-old) groups (Radavelli-Bagatini et al., 2021; Rubega et al., 2021).

All human-related study procedures were launched in compliance with the ethical guidelines of the institutional and/or national research committee, as well as the 1964 Declaration of Helsinki and its subsequent revisions or equivalent ethical standards. This research obtained ethical approval and was implemented under the oversight of the ethical review board from the Hospital (Human Investigation Committee PJKT2023-014). The trial registration number is ChiCTR2300069872. Clinical data such as medical history, age, sex, blood pressure, glucose (GLU), blood lipids, homocysteine (HCY), triglyceride (TG), cholesterol (CHOL), lipoprotein phospholipase A (LPA), and IMT were collected. Hypertension was diagnosed when patients were presented with resting blood pressure ≥140/90 mmHg or take antihypertensive medications. A fasting blood glucose level ≥6.1 mmol/L or an HbA1c ≥ 7% was defined as diabetes mellitus.

### 2.2 Measurement of vascular ageing and CAS

B-mode ultrasonography (Karnati et al., 2026) was employed to quantify carotid intima-media thickness (cIMT). Participants lay supine, neck extended and head rotated ~45°away from the side being examined. Images were acquired at the far wall of the distal common carotid artery (CCA) by specifically targeting a segment 1 cm proximal to the carotid bifurcation. Three consecutive measurements were acquired bilaterally for each participant, and the mean cIMT value per individual was calculated for subsequent analysis. Image acquisition deliberately excluded arterial segments exhibiting sonographic evidence of atherosclerotic plaques. An IMT > 0.58 mm indicates VA (Engelen et al., 2013; Andreassi et al., 2015; Hamczyk et al., 2020). All of the ultrasound examinations were conducted by experienced, certified sonographers unaware of the participants’ clinical details.

Carotid atherosclerosis was assessed by using carotid computed tomography angiography (CTA) with a 64-slice multidetector CT scanner (Philips, United States). During image acquisition, participants lay supine with their head fixed in place. An iodinated contrast agent (80–100 mL, 300–370 mgI/mL) was injected into the antecubital vein at 4–5 mL/s. Scanning was initiated when the aortic arch reached 100 HU. Images were reconstructed at 1-mm thickness and analysed by using axial, multiplanar, and 3D reconstructions (Annoni et al., 2019). The diverse degree of stenosis was categorized as mild (<50%), moderate (50–69%), or severe (≥70% or occlusion) (Barnett et al., 1998). Two experienced radiologists masked to clinical information, independently reviewed all measurements.

### 2.3 CircRNA sequencing

CircRNA sequencing data were analysed as per the manufacturer’s guidelines (Nielsen et al., 2022). Total circRNA from EMVs of healthy controls, VA participants and CAS patients (*n* = 3/group) was isolated by using the QIAGEN miRNeasy Micro Kit (QIAGEN) in accordance with the protocols. RNA purity was assessed by A260/A280 ratio using a NanoDrop 1,000 spectrophotometer. The concentration and integrity of the extracted total RNA were assessed by Agilent 2,100 Bioanalyzer (Applied Biosystems, Carlsbad, CA). RNA sequencing libraries were generated by using approximately 1 μg of total RNA with the KAPA RNA HyperPrep Kit with RiboErase (HMR) for Illumina® (Kapa Biosystems, Inc., Woburn, MA). Selective depletion of ribosomal RNA from the total RNA was performed. The RNA was fragmented and then sequentially subjected to first-strand synthesis and directional second-strand synthesis. Following this step, polyadenylation and adapter ligation was conducted on purified cDNA molecules. cDNA fragments coupled with sequencing adapters were further amplified via PCR to construct the ultimate sequencing library. The overall purity and yield of finished libraries were determined using a DNA 1000 chip on the Agilent 2,100 Bioanalyzer system. Accurate library quantitation ahead of sequencing was accomplished with the qPCR-based quantification kit (Kapa Biosystems). All prepared libraries were normalized to a terminal concentration of 10 nM and blended at equal molar ratios for subsequent cluster generation. Subsequently, all of the samples underwent 150 bp paired-end (PE150) sequencing. The detailed bioinformatics analysis could be found in the Supplementary materials.

### 2.4 EMVs isolation

Circulating EMVs were isolated and characterized as reported in our previous study (Zhang et al., 2020). EMVs were isolated via sequential centrifugation and immunomagnetic bead-based enrichment using anti-CD105 and anti-CD144 antibodies, followed by Q-dot 655 labeling. Detailed experimental procedures are provided in the Supplementary materials.

### 2.5 EMVs characterization

The characterization of EMVs was conducted by using nanoparticle tracking analysis (NTA), transmission electron microscopy (TEM) andwestern blotting to determine their size, concentration, and morphology. The NanoSight NS300 system (Malvern Panalytical) was applied to measure the quantity and size distribution of the EMVs (Zhang et al., 2020). EMVs were diluted in 1 mL of sterile PBS to optimize the particle concentration for analysis. The accompanying software of NTA automatically calculated the mean particle size and concentration.

The vesicle marker Annexin V was examined via western blotting. The proteins were separated by using 12% SDS-PAGE gels and subsequently transferred onto polyvinylidene difluoride (PVDF) membranes (pore size: 0.22 mm; Millipore, Billerica, United States). To prevent nonspecific binding, membranes were blocked with 5% skim milk for 1 h prior to incubation with anti-GAPDH (1:1000; Abcam, cat# ab8245) and Annexin V (1:1000; Abcam, cat# ab313581) primary antibodies. Protein detection was performed by using chemiluminescence imaging on the Azure C600 system (Azure Biosystems, United States). Relative abundance of target proteins was quantified with ImageJ software (NIH), with GAPDH serving as a control for normalization. All experiments were performed in triplicate. GraphPad Prism 5 was used for statistical analysis.

Morphological characterization of EMVs was conducted via TEM (Zhang et al., 2020). Briefly, After air-drying a 10 μL EMVs suspension drop on a carbon-coated copper grid at room temperature, the grid was visualized under a JEM-1400 TEM (Hitachi, Japan) operated at 80 kV. High-resolution micrographs were captured by using a digital camera to visualize the sizes and shapes of the EMVs.

### 2.6 Analysis of plasma EMVs-circ_0006222

To further characterize CD105^+^ EMVs, they were incubated with anti-CD144-conjugated beads to obtain CD105^+^CD144^+^ EMVs (Zhang et al., 2020). Total RNA was extracted from 200 μL of the EMV samples by using the TRIzol in combination with the miRNeasy Mini Kit (QIAGEN, Hilden, Germany). The purified RNA was collected and stored at −80 °C. Detailed information is available in the Supplementary materials.

EMVs samples was reversely transcribedinto complementary DNA (cDNA) templates, utilizing the Evo M-MLV Reverse Transcriptase Kit (Accurate Biology, Hunan, China) following the protocols. Reverse transcription and quantitative real-time PCR (qRT-PCR) were performed to assess the levels of circ_0006222 in the EMVs. The primers used for amplification were from GenePharma (Suzhou, China). The expression level of EMVs-circ_0006222 was quantified by using the 2^−△△Ct^ method, with U6 being used as control for normalisation. The following specific primers for circ_0006222 were used for qRT-PCR: forward, 5′-GAC ATG TTA GTA CAG CAG AAG AGA TG-3′; and reverse, 5′-TGT CCT TGC CTG GAA GAT AAA-3′. Quantification of EMVs-circ_0006222 was performed by two investigators who were masked to the clinical information of the participants. Detailed information is available in the Supplementary materials.

### 2.7 Statistical analysis

All statistical computations were conducted on GraphPad Prism 7.0 and SPSS 26.0 software. Continuous clinical variables were expressed as mean ± standard deviation (SD) or as median with interquartile range (IQR). Proportions were compared using Pearson’s test. The Kolmogorov–Smirnov test was used to examine the normality of the data. Plasma EMVs-circ_0006222 levels between the VA and CAS groups were compared by using the Mann–Whitney test. Differences in plasma EMVs-circ_0006222 levels among different stages of age and severity of CAS were analysed via the Kruskal Wallis test. The correlation between plasma EMVs-circ_0006222 levels and age was assessed by using Spearman correlation analysis. The relationship between EMVs-circ_0006222 levels and CAS was analysed via logistic regression. ROC curves and AUCs were used to test the sensitivity and specificity of the EMVs, EMVs-circ_0006222 and their combined measurements to compare their ability to diagnose VA and CAS. Differences exhibiting *p* < 0.05 were regarded as statistically significant.

## 3 Results

### 3.1 General characteristics of the study population

The baseline demographic features of all enrolled subjects were listed in Supplementary Table 1. A total of 47 healthy controls, 81 VA participants, and 216 patients with CAS were enrolled in the study. Age, systolic blood pressure (SBP), diastolic blood pressure (DBP), HCY, and TGs were significantly greater in patients with VA and CAS than in controls. No differences were observed in sex, GLU, CHOL, and LPA among the three groups. The proportions of hypertension and diabetes mellitus histories demonstrated significant differences. Moreover, no significant difference was detected between VA and CAS regarding age, SBP, DBP, HCY and TG. LPA and CHOL did not differ among the three groups. Baseline data of controls, VA patients, and subjects with non-CAS or CAS at different ages are illustrated in Supplementary Tables 2, 3.

### 3.2 Determination of isolated EMVs

As presented in Supplementary Figure 1, NTA and TEM characterization demonstrated the particle diameter of isolated EMVs exhibited a diameter distribution spanning between 100 and 700 nm (Supplementary Figure S1A, B). Immunoblotting confirmed the presence of Annexin V, which is a marker for microvesicles in EMVs (Supplementary Figure S1C).

### 3.3 Distribution and diversity of circulating EMV-circRNAs

**Figure 1:**
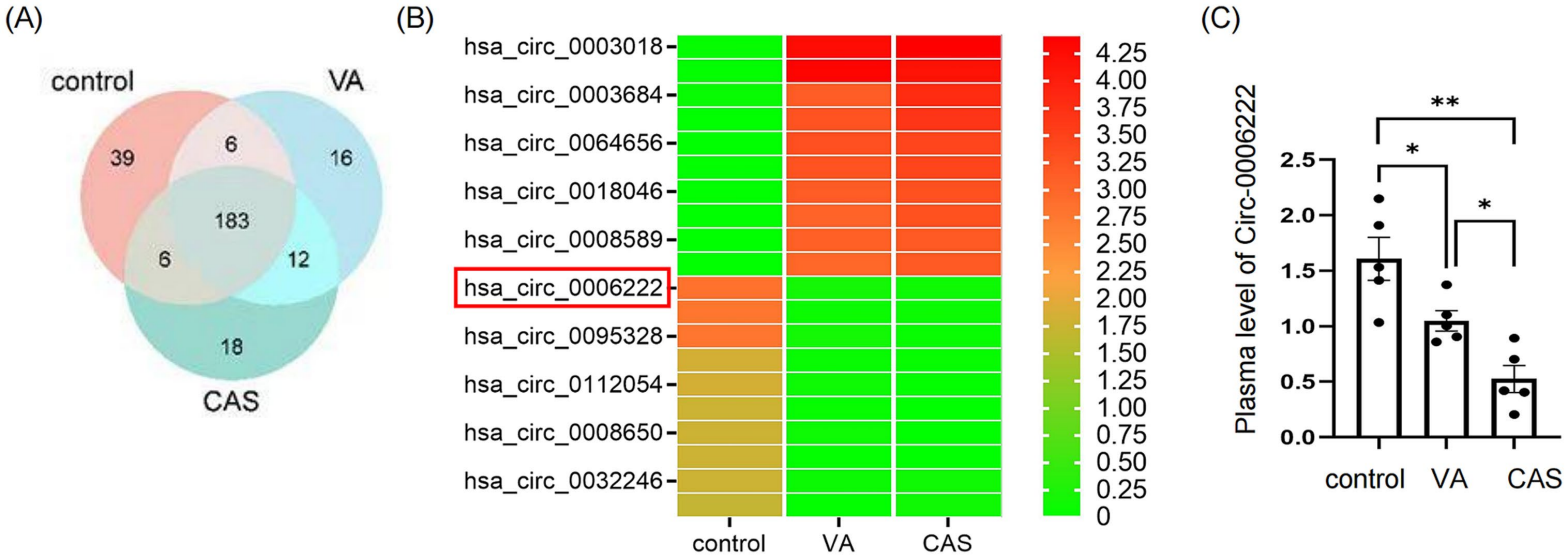
Heatmap illustrating the top ten differentially expressed circRNAs (both up- and down-regulated) in EMVs from control, VA and CAS participants. **(A)** Distribution and diversity of circulating EMV-circRNAs analyzed by high-throughput transcriptome sequencing. **(B)** Scatter plot with axes showing the mean normalized signal values (log2 scaled) of the sample groups. Rows represent circRNAs and columns represent samples. Red and green denote upregulation and downregulation, respectively. **(C)** EMVs-circ_0006222 levels in the control, VA and CAS groups, as assessed via qRT-PCR (control vs. VA: ^*^p = 0.04; control *vs*. CAS: ^**^p = 0.002; VA vs. CAS: ^*^p = 0.045).

To screen the EMV-cicRNAs involved in the CAS process, we performed high-throughput transcriptome sequencing to profile EMV-circRNAs with differential expression in healthy controls, as well as in VA and CAS participants (*n* = 3/group). Total RNA was isolated from plasma EMVs and analysed via RNA sequencing. We performed differential expression analyses for VA versus control and CAS versus control. The intersection of these analyses revealed a common set of 183 circRNAs that were consistently differentially expressed in both VA and CAS (fold change ≥2.0 and *p* ≤ 0.05 in both comparisons). Within this common set, 115 circRNAs were commonly downregulated, and 68 circRNAs were commonly upregulated, compared with those in healthy controls (Figure 1A).

The ten circRNAs with the highest differential expression are presented in Figure 1B. To validate the sequencing findings, the levels of EMVs-circ_0006222 in healthy controls, as well as in VA and CAS participants (*n* = 5/group), were further confirmed via qRT-PCR. The data revealed that the level of EMVs-circ_0006222 was dramatically decreased in the VA and CAS groups, which aligned with the sequencing results (Figure 1C).

### 3.4 Plasma EMVs-circ_0006222 were negatively correlated with age

**Figure 2:**
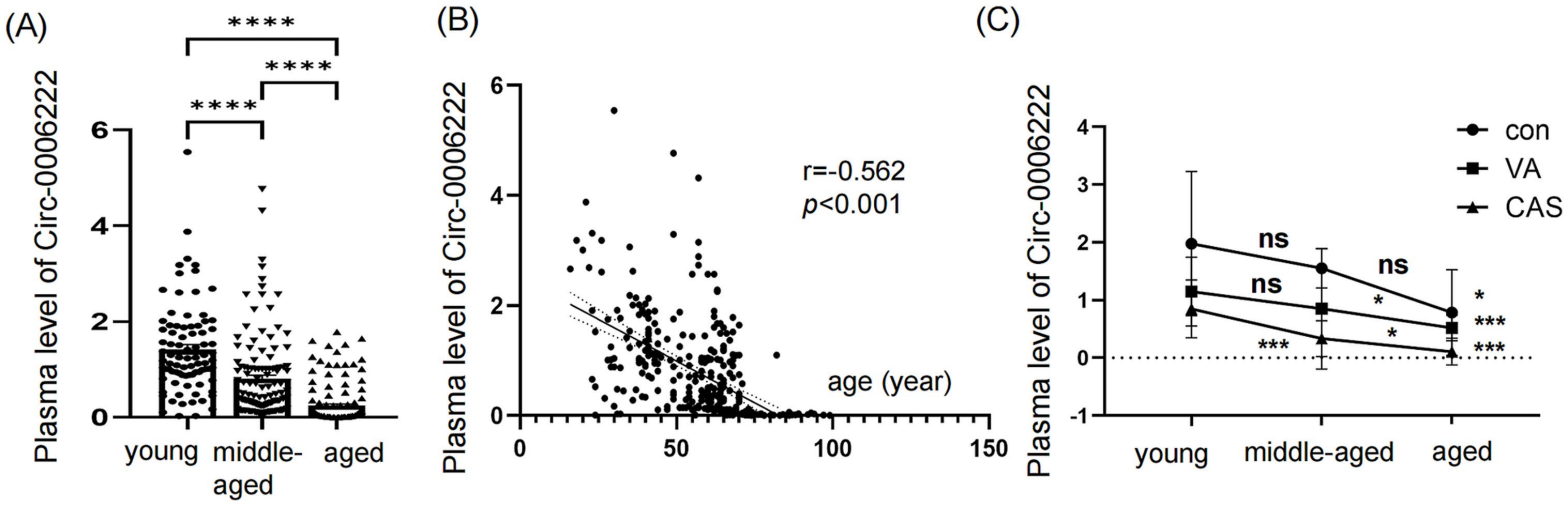
Plasma EMVs-circ_0006222 levels in various age groups and in both control, VA and CAS participants. **(A)** Plasma EMVs-circ_0006222 levels in young, middle-aged, and aged participants (^***^p < 0.001). **(B)** Spearman correlation analysis between plasma EMVs-circ_0006222 levels and age, demonstrating a significant negative correlation (*r* = −0.562, *p* < 0.001). **(C)** Comparison of EMVs-circ_0006222 levels across different age groups among the control, VA and CAS participants (control group: young vs. middle-aged participants, no significant difference (*ns*); middle-aged vs. aged participants, *ns*; young vs. aged participants, ^*^p = 0.02; VA group: young vs. middle-aged participants, *ns*; middle-aged *vs*. aged participants, ^*^p = 0.041; young vs. aged participants, ^***^p < 0.001; CAS group: young *vs*. middle-aged participants, ^***^p < 0.001; middle-aged *vs*. aged participants, ^*^p = 0.045; young vs. aged participants, ^***^p < 0.001).

A marked decrease in plasma EMVs-circ_0006222 levels was observed in aged participants compared with young and middle-aged ones (young versus middle-aged participants: 1.42 ± 0.10 versus 0.80 ± 0.08, *p* < 0.001; young versus aged participants: 1.42 ± 0.10 versus 0.24 ± 0.04, *p* < 0.001; middle-aged versus aged participants: 0.80 ± 0.08 versus 0.24 ± 0.04, p < 0.001; Figure 2A). In all of the participants, a significant inverse relationship was observed between EMVs-circ_0006222 and age. (*r* = −0.562, *p* < 0.001, Figure 2B).

We further investigated the age-related changes in EMVs-circ_0006222 levels among the healthy control, VA, and CAS groups. The results revealed that plasma EMVs-circ_0006222 levels decreased with increasing age in all three groups (Figure 2C). In the healthy controls, no statistical differences were detected in EMVs-circ_0006222 levels. Between the young, middle-aged subjects or between the middle-aged and the elderly subjects. In the VA group, the EMVs-circ_0006222 level did not significantly differ between the young group and the middle-aged group; however, this level was distinctly lower in the aged group than in the middle-aged group (0.52 ± 0.09 vs. 0.87 ± 0.52, *p* = 0.041; Figure 2C). In both the healthy control and VA groups, a marked decrease in EMVs-circ_0006222 levels were found in the aged group compared with the young group (control: 1.98 ± 0.23 vs. 0.78 ± 0.26, *p* = 0.02; VA: 1.15 ± 0.16 vs. 0.52 ± 0.09, *p* < 0.001; Figure 2C). In the CAS group, the plasma EMVs-circ-0006222 level was observed to be the lowest in the aged group, compared with that in the middle-aged and young groups (young vs. middle-aged groups: 1.15 ± 0.07 vs. 0.34 ± 0.04, p < 0.001; young vs. aged groups: 1.15 ± 0.07 vs. 0.11 ± 0.02, p < 0.001; middle-aged vs. aged groups: 0.34 ± 0.04 vs. 0.11 ± 0.02, *p* = 0.045; Figure 2C); moreover, differences comparing the young with the middle-aged, and the middle-aged with the aged, also demonstrated statistical significance.

### 3.5 Plasma EMVs-circ_0006222 concentrations were substantially decreased in the VA and CAS groups, with a more marked reduction in the severe CAS group

**Figure 3:**
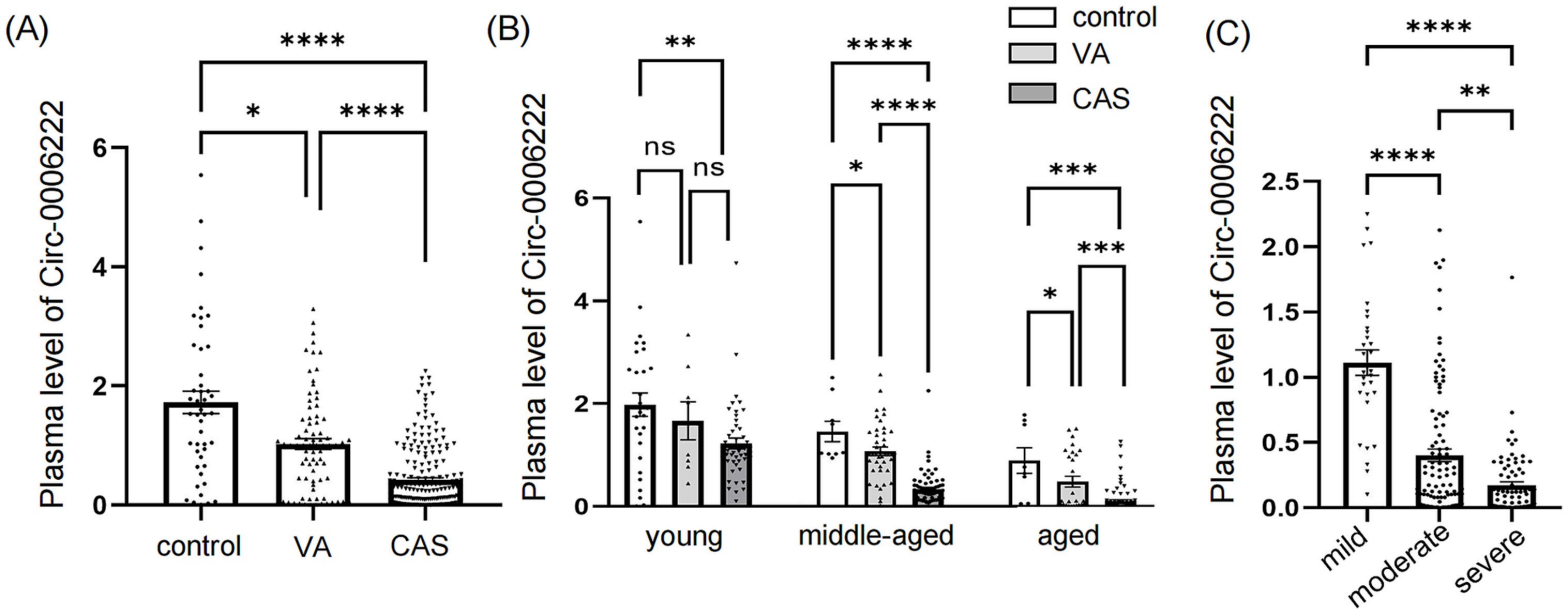
Plasma EMVs-circ_000622 levels in control, VA and CAS participants and different severity of CAS. **(A)** Comparison of plasma EMVs-circ_0006222 levels among control, VA and CAS participants (control vs. VA: ^*^p = 0.049; VA vs. CAS: ^***^p < 0.001; control vs. CAS: ^***^p < 0.001). **(B)** Plasma EMVs-circ_0006222 levels in control, VA and CAS participants across different age groups (young group: control vs. VA, *ns*; VA vs. CSA, *ns*; control vs. CAS, ^**^p = 0.003; middle-aged group: control vs. VA, ^*^p = 0.04; VA *vs*. CSA, ^***^p < 0.001; control *vs*. CAS, ^***^p < 0.001; aged group: control *vs*. VA, ^*^p = 0.02; VA *vs*. CSA, ^***^p < 0.001; control vs. CAS, ^***^p < 0.001). **(C)** Plasma EMVs-circ_0006222 levels in different CAS severity groups (mild *vs*. moderate: ^***^p < 0.001; mild vs. severe: ^***^p < 0.001; moderate *vs*. severe: ^**^p = 0.009).

The data demonstrated that the plasma EMVs-circ_0006222 levels were significantly reduced in the VA and CAS participants compared to control individuals (control vs. VA: 1.73 ± 1.29 vs. 1.03 ± 0.82, *p* = 0.049; control vs. CAS: 1.73 ± 1.29 versus 0.42 ± 0.53, *p* < 0.001; Figure 3A). The plasma EMVs-circ_0006222 level in the CAS group decreased by approximately 6-fold compared to the VA group (1.03 ± 0.82 vs. 0.42 ± 0.53, *p* < 0.001; Figure 3A).

The EMVs-circ_0006222 levels among the control, VA, and CAS groups were compared across different age groups. The results revealed that in young participants, the plasma levels of EMVs-circ_0006222 were obviously lower in the CAS group than in the healthy controls (1.99 ± 0.23 vs. 1.22 ± 0.10, *p* = 0.003; Figure 3B), and no notable statistical differences observed in EMVs-circ_0006222 level between the control and the VA groups (1.99 ± 0.23 vs. 1.66 ± 0.36, *p* = 0.71; Figure 3B) or between the VA and the CAS groups (1.66 ± 0.36 vs. 1.22 ± 0.10, *p* = 0.46; Figure 3B). In the middle-aged group, the plasma levels of EMVs-circ_000622 were significantly decreased in the VA and CAS groups than in the healthy controls (control vs. VA: 1.46 ± 0.20 vs. 1.06 ± 0.08, *p* = 0.04; control vs. CAS: 1.46 ± 0.20 vs. 0.32 ± 0.03, *p* < 0.001; Figure 3B), and statistical difference was shown between the VA and CAS groups (1.06 ± 0.08 vs. 0.32 ± 0.03, *p* < 0.001; Figure 3B). In the aged group, the plasma EMVs-circ_0006222 levels demonstrated a progressive decrease in the VA and CAS groups, and the reduction was statistically more prominent in the CAS group (control vs. VA: 0.89 ± 0.25 vs. 0.47 ± 0.10, *p* = 0.02; control vs. CAS: 0.89 ± 0.25 vs. 0.10 ± 0.03, *p* < 0.001; VA vs. CAS: 0.47 ± 0.10 vs. 0.10 ± 0.03, *p* < 0.001; Figure 3B).

We subsequently examined the relationship between the plasma level of EMVs-circ_0006222 and the different severity of CAS. The results revealed that the plasma level of EMVs-circ_0006222 was markedly lower in the moderate group (mild vs. moderate: 1.11 ± 0.54 vs. 0.40 ± 0.49, *p* < 0.0001; Figure 3C) and the severe group (mild vs. severe: 1.11 ± 0.54 vs. 0.17 ± 0.25, *p* < 0.0001; Figure 3C) than in the mild group. Significant statistical difference also existed between the moderate and severe groups. (moderate vs. severe: 0.17 ± 0.25 vs. 1.11 ± 0.54, *p* = 0.009; Figure 3C).

### 3.6 Plasma EMVs-circ_0006222 was an independent and favourable diagnostic biomarker for VA and CAS

We applied multivariate logistic regression analysis to investigate the relationships between EMVs-circ_0006222 and both CAS and VA. The results indicated that age (OR = 0.98, 95% CI: 0.96–1.00), SBP (OR = 1.00, 95% CI: 0.99–1.02), Glu (OR = 1.28, 95% CI: 1.06–1.55), and EMVs-circ_0006222 (OR = 0.19, 95% CI: 0.12–0.31) were associated with VA (Tables 1, 2). Age (OR = 1.05, 95% CI: 1.02–1.07), SBP (OR = 1.02, 95% CI: 1.00–1.03), Glu (OR = 1.35, 95% CI: 1.09–1.67), HCY (OR = 1.15, 95% CI: 1.07–1.24) and EMVs-circ_0006222 (OR = 0.19, 95% CI: 0.11–0.33) were associated with CAS.

**Table 1:** Multivariate logistic regression between VA and risk factors.

Items	OR	95% CI	*p*-value
Age	0.98	0.96–1.00	0.05
SBP	1.00	0.99–1.02	0.12
Glu	1.28	1.06–1.55	0.01
EMVs-circ_0006222	0.19	0.12–0.31	<0.001

**Table 2:** Multivariate logistic regression between CAS and risk factors.

Items	OR	95% CI	*p*-value
Age	1.05	1.02–1.07	<0.001
SBP	1.02	1.00–1.03	0.02
Glu	1.35	1.09–1.67	0.006
HCY	1.15	1.07–1.24	<0.001
EMVs-circ_0006222	0.19	0.11–0.33	<0.001

**Figure 4:**
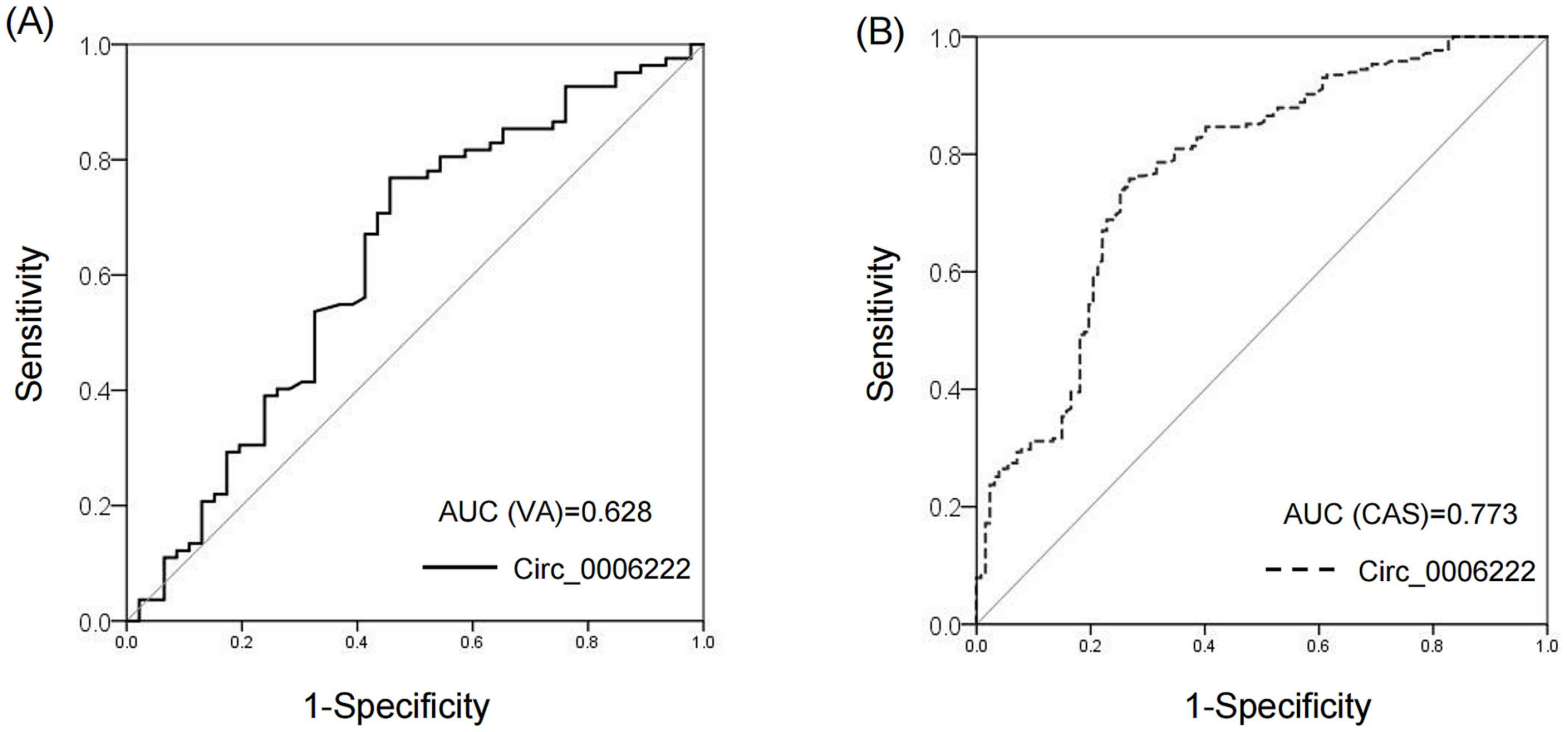
ROC curves for discriminating VA and CAS by EMVs-circ_0006222. **(A,B)** Plasma EMVs-circ_0006222 exhibited relatively high diagnostic accuracy for VA and CAS (VA: AUC = 0.628; specificity, 54.3%; sensitivity, 76.8%; CAS: AUC = 0.773; specificity, 73.2%; sensitivity, 75.8%).

To assess the diagnostic value of EMVs-circ_0006222 for VA and CAS, ROC curves were constructed to discriminate VA and CAS subjects from non-VA and non-CAS participants. As shown in Figure 4, the AUC revealed that plasma EMVs-circ_0006222 could serve as valuable biomarkers for VA and CAS, with AUCs of 0.628 (95% CI: 0.523–0.733, specificity: 54.3%, sensitivity: 76.8%, *p* = 0.017) and 0.773 (95% CI: 0.720–0.825, specificity: 73.2%, sensitivity: 75.8%, *p* < 0.001) being observed, respectively. The diagnostic thresholds of EMVs-circ_0006222 in the VA and CAS groups were 1.51 nmol/L and 0.639 nmol/L, respectively (Tables 3, 4).

**Table 3:** AUC, sensitivity, specificity, cutoff levels for EMVs-circ_0006222 in VA.

Items	AUC	Cut off value	Sensitivity	Specificity	Youden index	95% CI	*p-*value
EMVs-circ_0006222	0.628	<1.51	76.8%	54.3%	0.312	0.523–0.733	0.017

**Table 4:** AUC, sensitivity, specificity, cutoff levels for EMVs-circ_0006222 in CAS.

Items	AUC	Cut off value	Sensitivity	Specificity	Youden index	95% CI	*p*-value
EMVs-circ_0006222	0.773	<0.639	75.8%	73.2%	0.409	0.720–0.825	<0.001

## 4 Discussion

This study demonstrated that plasma levels of EMVs-circ_0006222 were inversely correlated with age and were significantly decreased in VA and CAS participants. The reduction was more pronounced in older participants with VA and CAS and in those with more severe CAS. The plasma EMVs-circ_0006222 level was observed to be an independent factor for VA and CAS and demonstrated diagnostic value. These findings illustrate that plasma EMVs-circ_0006222 may act as promising biomarker for VA and CAS. Notably, this research represents an unprecedented investigation of plasma EMVs-circ_0006222 levels in patients with VA and CAS.

VA and CAS represent systemic arterial dysfunction and focal atherosclerosis, respectively, and their progression is often asymptomatic until a major cerebrovascular event occurs, thus highlighting the vital necessity for early detection strategies (Wang et al., 2025). The current diagnostic paradigm mainly depends on imaging methods such as carotid Doppler ultrasound and can identify structural changes only when they are already at advanced stages (Engelen et al., 2013). The utility of molecular biomarkers, especially those focused on inflammatory pathways, may be compromised by nonvascular systemic conditions. EMVs that are released from endothelial cells are significantly increased secondary to inflammatory stimulation, oxidative stress, and coagulation disorders (Wang et al., 2025). Recent investigations have documented a robust relationship between EMVs and ageing, atherosclerosis, and both cardiovascular and cerebrovascular pathologies (Chmielewski et al., 2024). Compared with those in age-matched normal-weight and normotensive adults, plasma EMVs levels are increased in hypertensive patients and obese hypertensive patients (Fandl et al., 2023). Our previous study and other researches have revealed that plasma EMVs levels are significantly elevated in patients with acute cerebral infarction and are positively correlated with IMT, atherosclerosis progression, NIHSS scores and infarct volume (Huo et al., 2021; Ma et al., 2023). CircRNAs are abundant, conserved, and tissue-specific noncoding RNAs that play key roles in vascular biological processes (Jakobi, 2025). Because of their exceptional stability in the circulation and resistance to RNase degradation, circRNAs display expression patterns that are highly cell-type- and disease-state-specific. A recent study indicated that plasma hsa-circ-0105015 levels are increased in essential hypertension patients and are correlated with vascular inflammation and endothelial dysfunction (He et al., 2021). Circ-HIPK3 is highly expressed in vulnerable atherosclerotic plaques obtained from patients undergoing carotid endarterectomy surgery, and circ-HIPK3 induces ROS production in vascular smooth muscle cells (Li et al., 2025). Studies have displayed that 25 differentially expressed circRNAs have been validated in patients with large artery atherosclerosis stroke and normal controls; moreover, exosomal circ_001015 and circ_0005585 could serve as potential diagnostic biomarkers (Xiao et al., 2021). Elevated plasma levels of circ_0043621 have demonstrated diagnostic value in discriminating carotid plaque patients from controls (Yan et al., 2024). These studies emphasize the role of EMVs and circRNAs in inflammatory processes and vascular pathology. Chronic inflammation and increased oxidative stress levels in the injured endothelium are strongly involved in the early stage of ageing and atherosclerosis (Shao et al., 2024; You et al., 2025). Therefore, EMVs carrying circRNAs may play important roles in ageing and CAS, particularly given that circRNAs encapsulated in extracellular vesicles exhibit higher stability (Li et al., 2015). However, research exploring EMV-circRNAs as biomarkers for VA and CAS remains limited. Moreover, the search for reliable circRNA biomarkers packaged within EMVs for VA and CAS demonstrates significant clinical implications.

Our bioinformatics analysis revealed SENP1 as a target of circ-0006222. SENP1 plays important roles in various biological processes and cardiovascular-related diseases (Liu et al., 2024). Studies have demonstrated that SENP1 prevents endothelial cell apoptosis and myocardial ischaemia/reperfusion injury (Yang et al., 2022; Liu et al., 2024). SENP1-deficient mice exhibit elevated proinflammatory cytokine levels in the peripancreatic fat tissue, with direct cytotoxic effects being exerted on the pancreas (Shao et al., 2015). SENP1-overexpressing mouse embryonic fibroblasts can alleviate cellular senescence-induced ageing (Xia et al., 2016) and significantly suppress microglia-mediated inflammatory responses (Yang et al., 2020). Dysfunction in SENP1 transcriptional activity may lead to disruptions in the stress response and glucose-lipid metabolism, thereby triggering endothelial injury, VA, and atherogenesis (Pandey et al., 2018; Yu et al., 2020). These studies indicate that SENP1 is strongly involved in inflammatory events and represents a potential therapeutic target for VA and CAS. Our findings revealed that EMVs-circ_0006222 levels were reduced in the VA and CAS participants and were positively correlated with age and the degree of CAS. These results align with an observation demonstrating that symptomatic high-grade carotid plaques exhibit significantly higher inflammatory responses compared to asymptomatic plaques, as measured by serum levels of metalloproteinase-2 and metalloproteinase-9 (Alvarez et al., 2004). Among elderly patients, plaques from symptomatic individuals exhibited higher lipid and macrophage contents than those from asymptomatic individuals (Grufman et al., 2014). Previous studies have demonstrated that traditional inflammatory biomarkers such as C-reactive protein, IL-1, and IL-6 grew in parallel with increasing plaque severity (Ghelani and Alaour, 2026). These studies reveal that greater CAS severity and advanced age are associated with elevated oxidative stress levels and endothelial inflammation (El Assar et al., 2022). The altered levels of EMVs-circ_0006222 may serve as a novel molecular readout of oxidative stress and endothelial dysfunction in VA and CAS. Regression analysis revealed an independent association between EMVs-circ_0006222 levels and both VA and CAS; moreover, EMVs-circ_0006222 acted as a protective factor. ROC analysis revealed that EMVs-circ_0006222 could serve as a promising biomarker for VA and CAS. We suggest that EMVs-circ_0006222 may act as miRNA sponges to compete with miRNAs for binding to SENP1 mRNA transcripts, thus consequently affecting miRNA-mRNA interactions. However, this study has several limitations. For example, the mechanisms underlying the role of EMVs-circ_0006222 in vascular injury require further explorations. Additional studies with expanded, multicentre cohorts are essential to confirm the utility of EMVs-circ_0006222 across different ethnic and clinical subgroups.

## 5 Conclusion

Our findings demonstrate that plasma EMVs-circ_0006222 are downregulated in VA and CAS. Plasma EMVs-circ_0006222 exhibit considerable potential in the diagnoses of VA and CAS.

## Data Availability

The original contributions presented in the study are included in the article/Supplementary material, further inquiries can be directed to the corresponding authors.
