# Supplementary material for: Biomarker value of plasma endothelial microvesicle-derived circRNA 0006222 in vascular ageing and carotid atherosclerosis
Source: Front Neurosci. 2026 Jul 15;20:1872315. doi: 10.3389/fnins.2026.1872315 (PMC13415766; doi:10.3389/fnins.2026.1872315)
Supplement: Supplementary file 1 [file Table_1.DOCX]

**Supplementary table 1. Baseline characteristics of the participants**

|  | **CON (n=47)** | **VA (n=81)** | **CAS (n=216)** | ***p* value** |
| --- | --- | --- | --- | --- |
|  |  |  |  |  |
| Age, years | 35.(26-60) | 61 (55-66) | 64 (49-73) | <0.001 |
| Male sex | 26 (55.3%） | 39 (48.1%) | 103 (47.7%) | 0.53 |
| Female sex | 20 (42.5%） | 43 (53.1%) | 113 (52.3%) | 0.53 |
| Hypertension | 3 (6.4%） | 34 (41.9%) | 118 (54.6%) | <0.001 |
| DM | 4 (8.5%） | 10 (12.3%) | 47 (21.8%) | 0.04 |
| SBP, mmHg | 123 (112-136) | 138 (124-154) | 144 (129-161) | <0.001 |
| DBP, mmHg | 72 (66-80) | 80 (72-86) | 78 (69-85) | 0.007 |
| GLU, mmol/L | 5.0±1.1 | 5.3±1.7 | 5.6±1.9 | 0.05 |
| HCY, μmol/L | 7.0 (4.6-10.0) | 8.7 (5.4-12.2) | 9.7 (7.6-12.4) | <0.001 |
| TG, mmol/L | 1.3±0.7 | 1.4±0.9 | 1.7±0.9 | 0.03 |
| CHOL, mmol/L | 4.4±1.3 | 4.8±1.4 | 4.9±1.4 | 0.11 |
| LPA, mg/L | 131.1 (49.9±211.4) | 159.2 (77.2-259.1) | 141.8 (86.6-276.1) | 0.44 |

Comparison of demographic and clinical parameters across CON, VA and CAS groups. Values shown as mean ± standard deviation, median (IQR) and categorical variables as number (percentage). *p*<0.05 indicates statistical significance.

Abbreviations: CON: healthy controls; VA: vascular aging; CAS: carotid atherosclerosis; SBP, systolic blood pressure; DBP, diastolic blood pressure; DM, diabetes mellitus; GLU, glucose; HCY, homocysteine; TG, triglycerides; CHOL, total cholesterol; LPA, lipoprotein(a).

**Supplementary table 2. Baseline characteristics of controls and VA in different ages**

|  | **<45 years old** | | ***p* value** | **45-65 years old** | | ***p* value** | **>65 years old** | | ***p*value** |
| --- | --- | --- | --- | --- | --- | --- | --- | --- | --- |
|  | Con (n=30) | VA (n=8) |  | Con (n=9) | VA (n=42) |  | Con (n=8) | VA (n=31) |  |
| Age, years | 29±6.9 | 39±4.9 | <0.001 | 60±3.3 | 58±5.2 | 0.27 | 68 (68-71) | 68 (66-70) | 0.26 |
| Male sex | 20 (66.7%) | 5 (62.5%) | 0.7 | 6 (66.7%) | 28 (66.7%) | >.99 | 5 (62.5%) | 17 (54.8%) | >.99 |
| Female sex | 10 (33.3%) | 3 (37.5%) | 0.7 | 3 (33.3%) | 14 (33.3%) | >.99 | 3 (37.3%) | 14 (45.2%) | >.99 |
| Hypertension | 1 (3.3%) | 2 (25.0%) | 0.11 | 2 (22.2%) | 23 (54.8%) | 0.14 | 2 (25.0%) | 7 (22.6%) | >.99 |
| DM | 0 (0%) | 1 (12.5%) | 0.21 | 1 (11.1%) | 7 (16.7%) | 0.68 | 1 (12.5%) | 5 (16.1%) | >.99 |
| SBP, mmHg | 120 (111-130) | 130 (122-137) | 0.06 | 138 ±25.4 | 138±19.7 | 0.95 | 146 (105-155) | 137 (123-153) | 0.96 |
| DBP, mmHg | 72±9.9 | 74±6.1 | 0.53 | 73 (67-80) | 80 (75-88) | 0.04 | 72 (69-87) | 74 (69-83) | 0.99 |
| GLU, mmol/L | 4.7 (4.4-5.1) | 4.8 (4.5-5.3) | 0.30 | 4.8 (4.8-5.2) | 4.9 (4.6-5.8) | 0.88 | 4.9 (4.5-5.5) | 5.5 (4.5-6.0) | 0.11 |
| HCY, μmol/L | 6.1 (4.7-9.5) | 11.7 (6.7-20.5) | 0.04 | 10.2±1.7 | 10.2±2.9 | 0.96 | 3.8 (1.9-7.3) | 3.9 (2.4-7.9) | 0.52 |
| TG, mmol/L | 0.9 (0.6-1.3) | 1.7 (1.1-2.1) | 0.007 | 1.1 (1.0-1.5) | 1.3 (0.9-2.1) | 0.27 | 1.6±0.7 | 2.1±1.1 | 0.16 |
| CHOL, mmol/L | 4.4±1.2 | 4.5±0.9 | 0.88 | 4.4±1.4 | 5.0±1.4 | 0.26 | 4.2 (3.5-6.1) | 5.1 (3.8-5.5) | 0.80 |
| LPA, mg/L | 127.7 (41.9-365.4) | 83.9 (31.4-147.7) | 0.27 | 110.0 (58.6-147.2) | 144.2 (78.9-314.8) | 0.14 | 167 (132.8-202.0) | 181.3 (121.1-228.6) | 0.59 |

Baseline characteristics of healthy controls and VA in the young, middle-aged, and aged groups.

Continuous variables are presented as mean ± standard deviation, median (IQR) and categorical variables as number (percentage).

Abbreviations: VA, Vascular aging; SBP, systolic blood pressure; DBP, diastolic blood pressure; DM, diabetes mellitus; GLU, glucose; HCY, homocysteine; TG, triglycerides; CHOL, total cholesterol; LPA, lipoprotein(a).

**Supplementary table 3. Baseline characteristics of the participants with non-CAS or CAS in different ages**

|  | **<45 years old** | | ***p* value** | **45-65 years old** | | ***p* value** | **>65 years old** | | ***p* value** |
| --- | --- | --- | --- | --- | --- | --- | --- | --- | --- |
|  | Non-CAS (n=38) | CAS (n=49) |  | Non-CAS (n=51) | CAS (n=74) |  | Non-CAS (n=39) | CAS (n=93) |  |
| Age, years | 31 (24-36) | 40 (38-42) | <0.001 | 58 (54-61) | 61 (55-63) | 0.04 | 68 (66-70) | 74 (70-78) | <0.001 |
| Male sex | 25 (65.8%) | 28 (57.1%) | 0.41 | 34 (66.7%) | 41 (55.4%) | 0.21 | 22 (56.4%) | 63 (67.7%) | 0.21 |
| Female sex | 13 (34.2%) | 21 (42.9%) | 0.41 | 17 (33.3%) | 33 (44.6%) | 0.21 | 17 (43.6%) | 30 (32.3%) | 0.21 |
| Hypertension | 3 (8.8%) | 11 (22.4%) | 0.07 | 25 (49.0%) | 41 (55.4%) | 0.48 | 9 (23.1%) | 65 (69.9%) | <0.001 |
| DM | 1 (2.6%) | 2 (4.1%) | 0.71 | 8 (15.7%) | 16 (21.6%) | 0.41 | 6 (15.4%) | 27 (29.0%) | 0.09 |
| SBP, mmHg | 122 (112-133) | 129 (118-140) | <0.001 | 140 (124-156) | 150 (131-161) | 0.11 | 140 (123-152) | 154 (134-168) | 0.002 |
| DBP, mmHg | 72 (64-81) | 74 (66-80) | 0.51 | 80 (73-87) | 80 (71-87) | 0.52 | 75 (69-84) | 77 (69-86) | 0.68 |
| GLU, mmol/L | 4.7 (4.5-5.2) | 4.8 (4.2-5.2) | 0.31 | 4.8 (4.6-5.5) | 5.3 (4.7-5.9) | 0.04 | 5.0 (4.5-5.6) | 5.4 (4.7-6.4) | 0.03 |
| HCY, μmol/L | 6.6 (4.9-11.5) | 8.3 (5.9-10.8) | 0.23 | 10.2 (8.0-12.7) | 7.8 (9.5-12.0) | 0.60 | 4.2 (2.4-9.4) | 10.5 (8.9-13.5) | <0.001 |
| TG, mmol/L | 1.1 (0.7-1.6) | 1.0 (0.8-1.3) | 0.88 | 1.2 (1.0-1.9) | 1.2 (0.9-1.9) | 0.44 | 1.6 (1.0-2.2) | 1.5 (0.9-2.1) | 0.75 |
| CHOL, mmol/L | 4.6 (3.8-5.1) | 4.5 (4.1-5.4) | 0.38 | 4.8 (3.6-5.7) | 5.0 (4.2-6.1) | 0.24 | 4.8 (3.8-5.5) | 4.7 (3.6-5.3) | 0.77 |
| LPA, mg/L | 125.9 (40.6-249.4) | 137.2 (84.2-224.4) | 0.26 | 130.7 (77.3-298.0) | 124.4 (80.6-234.4) | 0.83 | 180.0 (122.0-221.0) | 176.3 (91.9-349.6) | 0.58 |

Baseline characteristics of non-CAS and CAS in the young, middle-aged, and aged groups. Continuous variables are presented as mean ± standard deviation, median (IQR) and categorical variables as number (percentage).

Abbreviations: CAS, carotid artery stenosis; SBP, systolic blood pressure; DBP, diastolic blood pressure; DM, diabetes mellitus; GLU, glucose; HCY, homocysteine; TG, triglycerides; CHOL, total cholesterol; LPA, lipoprotein(a).
