# Supplementary material for: Biomarker value of plasma endothelial microvesicle-derived circRNA 0006222 in vascular ageing and carotid atherosclerosis
Source: Front Neurosci. 2026 Jul 15;20:1872315. doi: 10.3389/fnins.2026.1872315 (PMC13415766; doi:10.3389/fnins.2026.1872315)
Supplement: Supplementary file 2 [file Data_Sheet_1.DOCX]

**Supplementary materials**

**The roles of plasma endothelial microvesicle-derived circRNA 0006222 in vascular ageing and carotid atherosclerosis**

Huiting Zhang^1†^, Ye Ye^2†^, Xiaobing Xu^1^, Dinghua Xu^1^, Jiawu Fu^1^, Yan Wang^3^, Yanfang Chen^3*^, Xiaotang Ma^1*^

1 Department of Neurology, Guangdong Key Laboratory of Age-Related Cardiac and Cerebral Diseases, Institute of Neurology, Affiliated Hospital of Guangdong Medical University, Zhanjiang, 524001, China

2 Guangdong Mental Health Center, Guangdong Provincial People's Hospital (Guangdong Academy of Medical Sciences), Southern Medical University, Guangzhou 510080, China

3 Second Affiliated Hospital of Guangdong Medical University, Zhanjiang, 524001, China

Correspondence: Xiaotang Ma and Yanfang Chen

Address: 57 South Renmin Road, Affiliated Hospital of Guangdong Medical University, Zhanjiang, 524001, China.

E-mail: Xiaotang Ma, [mxtgdmc@163.com](mailto:mxtgdmc@163.com)

**Bioinformatics analysis**

Raw sequencing reads were first subjected to quality control using FastQC (v0.11.9). Adapter sequences and low-quality bases (Q < 20) were trimmed using Trimmomatic (v0.39). Clean reads were aligned to the human reference genome (GRCh38/hg38) using STAR (v2.7.10a) with default parameters. Gene-level read counts were quantified using featureCounts (v2.0.3) against the GENCODE v35 annotation. Differential expression analysis of circRNAs between groups (healthy controls, VA participants, and CAS patients) was performed using the DESeq2 package (v1.38.3) in R (v4.2.0). CircRNAs with an absolute |log₂ fold change|≥ 1 and an adjusted P value < 0.05 (Benjamini–Hochberg correction for multiple testing) were considered differentially expressed. For circRNA identification, CIRCexplorer2 or CIRI2 was applied to detect back-splicing junctions from the STAR-aligned reads.

**EMV isolation**

Circulating EMVs were isolated and characterized as reported in our previous study ^[32]^. Plasma was isolated by centrifuging whole blood at 1500 g for 5 min (10 °C). The supernatant was then spun at 300 g (15 min), 2000 g (20 min), and 20,000 g (70 min) sequentially to collect total microvesicles (MVs) The precipitated MVs were immersed in 10 μL of biotin-conjugated anti-CD105 antibody (Miltenyi Biotec) at room temperature for 15 minutes, followed by 15 min of incubation with anti-biotin magnetic microbeads (Miltenyi Biotec). A DynaMag-2 magnetic separator (Life Technologies) was used to retrieve the microbead-labelled MVs. After isolation, the microbead‑bound MVs were incubated with multisort release reagent to separate the beads, yielding purified CD105⁺MVs. These purified MVs were subsequently labeled with anti‑CD144 antibody for 2 h, and then conjugated with Q‑dot 655 (1:350 dilution; Life Technologies) for an additional 2 h. The Q-dot 655-labelled particles were confirmed as EMVs. The obtained EMV pellet was resuspended in 100 μL of sterile, filtered phosphate-buffered saline (PBS) and stored at 4 °C for further processing.

**Analysis of plasma EMVs-circ_0006222**

To further characterize CD105^+^ endothelial microvesicles (EMVs), they were incubated with anti-CD144 conjugated beads to generate CD105^+^CD144^+^ EMVs. Total RNA was extracted from 200 μL of EMVs samples using TRIzol reagent (Invitrogen, Carlsbad, CA, USA) in combination with the miRNeasy Mini Kit (QIAGEN, Hilden, Germany). The EMVs were lysed by adding 1 mL of TRIzol reagent (Invitrogen, Carlsbad, CA) , followed by vigorous vortexing for 15 seconds. The samples were then incubated at room temperature for 5 minutes to ensure complete lysis. Subsequently, 140 μL of chloroform was added, and the tubes were manually inverted for 15 seconds to facilitate phase separation. The mixture was then incubated at room temperature for 3 minutes before being centrifuged at 12,000 × g for 15 minutes at 4°C. Following centrifugation, the upper aqueous phase was carefully transferred to a new RNase-free tube, and 1.5 volumes of absolute ethanol were added to facilitate RNA precipitation. The mixture was then applied to an RNeasy MiniElute spin column (QIAGEN, Hilden, Germany) inserted into a 2 mL collection tube. The column was centrifuged at 8,000×g for 1 minute at 4°C, and the flow-through was discarded. The remaining sample was loaded onto the same column, and the centrifugation step was repeated to ensure complete RNA binding. To remove contaminants, 700 μL of Buffer RWT was added in the sample, followed by centrifugation at 8,000×g for 30 seconds at 4°C. Then 500 μL of Buffer RPE was added and the mixture was centrifuged at 8,000×g for 15 seconds at 4°C. The supernatant was discarded and 500 μL of 80% ethanol was added, followed by centrifugation at 8,000×g for 2 minutes at 4°C. The RNeasy MiniElute spin column was transferred to a new 2 mL collection tube, the cap was opened, and the column was centrifuged at 12,000×g for 5 minutes at 4°C to remove residual ethanol. The column was then transferred to a new 1.5 mL RNase-free collection tube, and 14 μL of RNase-free water was added directly onto the membrane. The RNA was eluted by centrifugation at 12,000×g for 1 minute at 4°C, and the purified RNA was collected and stored at -80°C.

Reverse transcription and quantitative real-time PCR (qRT-PCR) were performed to assess the expression levels of circ-0006222 in extracellular microvesicles (EMVs). Primers for amplification were designed and synthesized by GenePharma (Suzhou, China).

Total RNA extracted from EMVs was reverse-transcribed into complementary DNA (cDNA) using the Evo M-MLV Reverse Transcriptase Kit (Accurate Biology, Hunan, China) following the manufacturer’s instructions. All procedures were performed on ice to minimize RNA degradation. The reverse transcription reaction mixture consisted of 2.0 μL of 5× RTase Reaction Buffer Mix II, 0.5 μL of Evo M-MLV RTase Enzyme, 1.2 μL of RT primer mixture (containing both target RNA RT primer and U6 RT primer), and 6.3 μL of RNA sample.The reaction was immediately carried out in a MasterCycler Gradient PCR system (Eppendorf, Germany). The reaction conditions were set as follows:37°C for 60 minutes,followed by 85°C for 5 minutes.

Quantitative reverse transcription PCR (qRT-PCR) was performed on a LightCycler480-II System (Roche Diagnostics, Penzberg, Germany) using the SYBR® Green Premix Pro Taq HS qPCR Kit (Accurate Biology, Hunan, China). Each reaction was carried out in a 10 μL system, consisting of 5.0 μL of 2× SYBR® Green Pro Taq HS Premix, 1.0 μL of template cDNA, 0.4 μL of primer mix, and 3.6 μL of DNase-free water. The qRT-PCR amplification was carried out under the following conditions: initial denaturation at 95°C for 3 minutes, followed by 40 cycles at 95°C for 5 seconds and 60°C for 30 seconds. The expression level of EMVs-circ_0006222 was quantified using the 2^−△△Ct^ method, with U6 as the internal control for normalization. The specific primers used for qRT-PCR were as follows:circ-0006222: forward, 5′-GAC ATG TTA GTA CAG CAG AAG AGA TG-3′, and reverse,5′-TGT CCT TGC CTG GAA GAT AAA-3′.The level of EMVs-circ_0006222 was analyzed independently by two investigators who were blinded to the clinical characteristics of the participants.
