# Supplementary figures and images for: Biomarker value of plasma endothelial microvesicle-derived circRNA 0006222 in vascular ageing and carotid atherosclerosis
Source: Front Neurosci. 2026 Jul 15;20:1872315. doi: 10.3389/fnins.2026.1872315 (PMC13415766; doi:10.3389/fnins.2026.1872315)

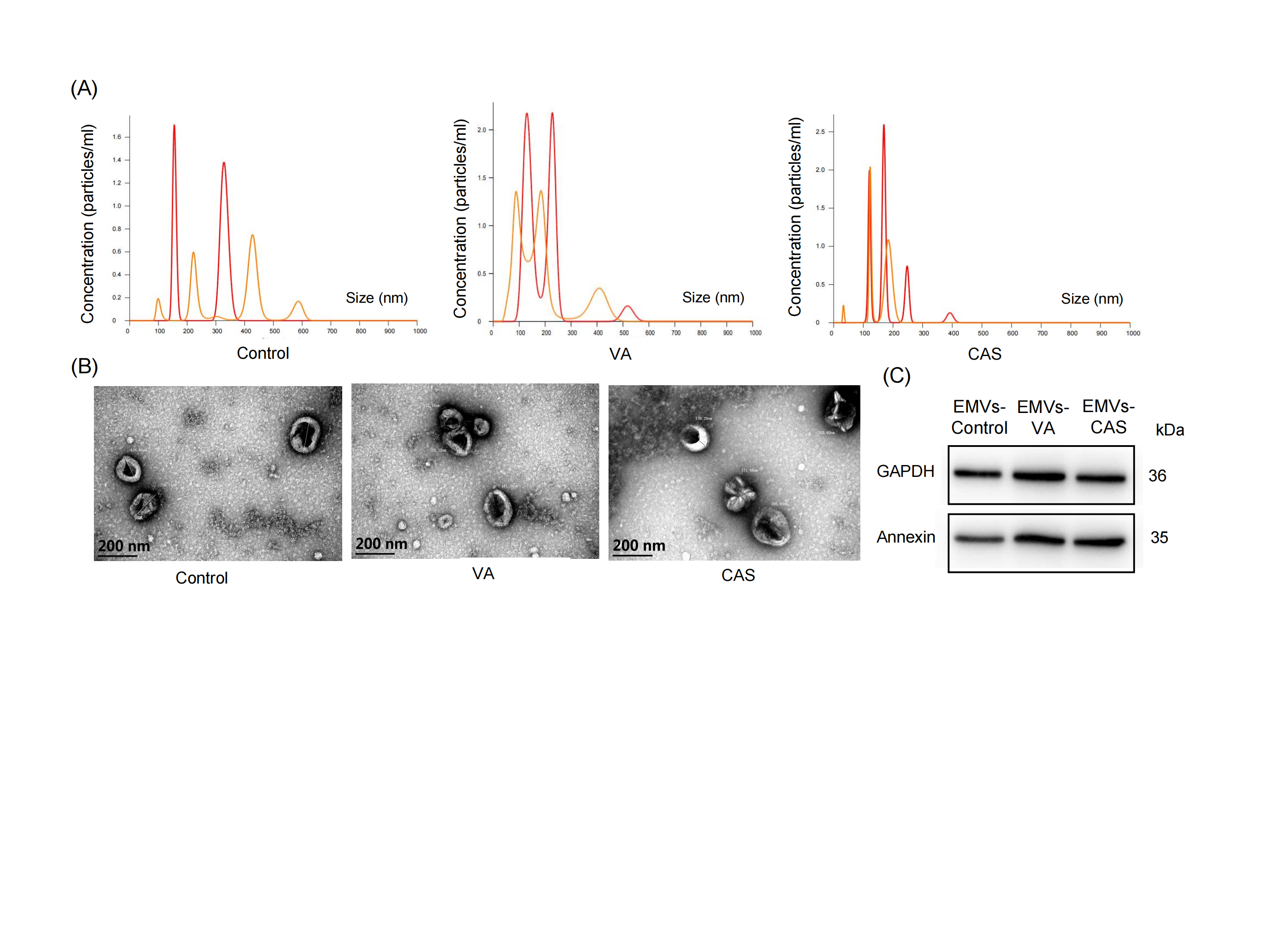

Supplement: Supplementary file 3 [file Image_1.TIF]
